# Sexually Dimorphic Patterns of Cell Proliferation in the Brain Are Linked to Seasonal Life-History Transitions in Red-Sided Garter Snakes

**DOI:** 10.3389/fnins.2018.00364

**Published:** 2018-06-01

**Authors:** Deborah I. Lutterschmidt, Ashley R. Lucas, Ritta A. Karam, Vicky T. Nguyen, Meghann R. Rasmussen

**Affiliations:** Department of Biology, Portland State University, Portland, OR, United States

**Keywords:** life-history stage, reproduction, courtship behavior, migration, dispersal, foraging, neurogenesis, reptile

## Abstract

Seasonal rhythms in physiology and behavior are widespread across diverse taxonomic groups and may be mediated by seasonal changes in neurogenesis, including cell proliferation, migration, and differentiation. We examined if cell proliferation in the brain is associated with the seasonal life-history transition from spring breeding to migration and summer foraging in a free-ranging population of red-sided garter snakes (*Thamnophis sirtalis*) in Manitoba, Canada. We used the thymidine analog 5-bromo-2′-deoxyuridine (BrdU) to label newly proliferated cells within the brain of adult snakes collected from the den during the mating season or from a road located along their migratory route. To assess rates of cell migration, we further categorized BrdU-labeled cells according to their location within the ventricular zone or parenchymal region of the nucleus sphericus (homolog of the amygdala), preoptic area/hypothalamus, septal nucleus, and cortex (homolog of the hippocampus). We found that cell proliferation and cell migration varied significantly with sex, the migratory status of snakes, and reproductive behavior in males. In most regions of interest, patterns of cell proliferation were sexually dimorphic, with males having significantly more BrdU-labeled cells than females prior to migration. However, during the initial stages of migration, females exhibited a significant increase in cell proliferation within the nucleus sphericus, hypothalamus, and septal nucleus, but not in any subregion of the cortex. In contrast, migrating males exhibited a significant increase in cell proliferation within the medial cortex but no other brain region. Because it is unlikely that the medial cortex plays a sexually dimorphic role in spatial memory during spring migration, we speculate that cell proliferation within the male medial cortex is associated with regulation of the hypothalamus-pituitary-adrenal axis. Finally, the only brain region where cell migration into the parenchymal region varied significantly with sex or migratory status was the hypothalamus. These results suggest that the migration of newly proliferated cells and/or the continued division of undifferentiated cells are activated earlier or to a greater extent in the hypothalamus. Our data suggest that sexually dimorphic changes in cell proliferation and cell migration in the adult brain may mediate sex differences in the timing of seasonal life-history transitions.

## Introduction

Seasonal changes in physiology and behavior are often associated with specific life-history stages, during which an organism is well suited to engage in particular functions within the appropriate environmental context. Examples of such life-history stages include reproduction, migration, parenting, foraging, and even territoriality vs. affiliative behavior. Critically, interactions between environmental conditions and resource availability can relegate life-history stages to a specific time of year and/or geographic locale. Transitions between life-history stages are therefore often characterized by dramatic behavioral changes that result from a shift in motivation to pursue one resource over another. While the mechanisms regulating life-history transitions are poorly understood, the concomitant changes in motivation suggest that a combination of mechanisms involving neuromodulation and neuroplasticity is likely.

One form of neuroplasticity that could contribute to seasonal changes in both appetitive and consummatory behaviors is neurogenesis. Post-natal neurogenesis is defined as the birth and maturation of new neurons that add to or replace neurons in the existing circuitry of the adult brain (e.g., Lindsey and Tropepe, 2006). Neurogenesis involves 5 different phases: cell proliferation within the ventricular zone (i.e., usually but not always within the ependymal layer of a ventricle), cell migration into the parenchyma, cell differentiation and maturation, cell integration into the existing synaptic network, and cell survival. It is hypothesized that seasonal changes in neurogenesis may regulate seasonal cycles in physiology and behavior (see review in Ebling, 2015). For example, both seasonal and circadian variation in cell proliferation, migration, and/or death has been described in many vertebrate and some invertebrate taxa (e.g., Goergen et al., 2002; Hansen and Schmidt, 2004; Vidal Pizarro et al., 2004; Dawley et al., 2006; Lindsey and Tropepe, 2006; Schmidt, 2007; Barnea and Pravosudov, 2011; Delgado-Gonzalez et al., 2011; Font et al., 2012; Smarr et al., 2014; Brenowitz, 2015; Sherry and MacDougall-Shackleton, 2015; Balthazart and Ball, 2016; Migaud et al., 2016; Akle et al., 2017; Yang et al., 2017). Moreover, there is now substantial evidence that the integration of new neurons into existing synaptic circuits in the adult brain contributes to both synaptic plasticity (e.g., long-term potentiation) and environmentally-induced plasticity (e.g., effects of enrichment and exercise) (Sahay et al., 2011; Singer et al., 2011; Alonso et al., 2012; Iscru et al., 2013; Patten et al., 2013; Darcy et al., 2014; Bergami et al., 2015; Temprana et al., 2015; Sakalem et al., 2017). Two questions that emerge prominently from these findings are: What function does such plasticity play in an animal's physiological and behavioral ecology, and how is neurogenesis regulated seasonally?

One of the most extensively studied functions associated with neurogenesis is its role in regulating learning and memory, including hippocampus-dependent spatial learning and memory (reviewed in Amrein and Lipp, 2009; Barnea and Pravosudov, 2011; Lieberwirth et al., 2016). Although early studies sometimes reported contradictory results regarding the association between neurogenesis and spatial learning (e.g., Nilsson et al., 1999; Meshi et al., 2006), it is now evident that the effects of neurogenesis depend on multiple processes, including the differential survival of relatively mature neurons, the death of relatively immature cells, and the timing of differentiation and integration of the surviving cells into existing synaptic circuits (Dupret et al., 2007; Farioli-Vecchioli et al., 2008). In an ecological context, neurogenesis-associated changes in spatial learning and memory have significant functional consequences for both survival and reproductive fitness. For example, in food-storing birds such as black capped chickadees (*Poecile atricapillus*), seasonal changes in neurogenesis and neuronal recruitment in the hippocampus are temporally linked to food-caching and retrieval behavior during the autumn and winter months (Hoshooley and Sherry, 2007; Hoshooley et al., 2007; Sherry and Hoshooley, 2010; Sherry and MacDougall-Shackleton, 2015). Increased neurogenesis appears to be critical to the process of spatial learning during food caching, as treatment of birds with the cell proliferation inhibitor methylazoxymethanol significantly decreased their performance on a spatial learning task (Hall et al., 2014). Spatial learning and memory is also critical to the process of migrating from one geographic locale to another, especially in animals that are faithful to a particular breeding territory, hibernacula, or foraging site. Several studies suggest that seasonal changes in hippocampal neurogenesis are associated with migratory behavior in birds (LaDage et al., 2011; Barkan et al., 2014; also see review in Barnea and Pravosudov, 2011). Whether a similar association between neurogenesis and seasonal migration exists in other vertebrate groups has not been directly examined. However, several key studies indicate that variation in hippocampal neurogenesis and/or volume is indeed positively correlated with the spatial memory demands of defending territorial boundaries, establishing social hierarchies, and determining home range size in ectotherms (Roth et al., 2006; LaDage et al., 2009, 2013; Holding et al., 2012, also see review in Powers, 2016).

Because spatial learning and memory is regulated by the hippocampus, a majority of studies on the function of neurogenesis have necessarily focused on cell proliferation, migration, differentiation, and integration within this region of the brain. This is especially true in mammals, where the regions of proliferative activity are limited to the subventricular zone of the lateral ventricles and the subgranular zone of the dentate gyrus within the hippocampus. In non-mammalian animals, however, the sites of neurogenesis are more widespread throughout the brain, and the rate of cell proliferation within these brain regions is much greater than that in mammals (Lindsey and Tropepe, 2006; Kaslin et al., 2008). Thus, we have relatively little information about the potential role of neurogenesis in other neurogenic brain regions, including the hypothalamus and amygdala, which are important in modulating reproductive and social behaviors. An additional area of complexity in understanding the role of neurogenesis is the existence of sex differences in both basal rates of cell proliferation, migration, and differentiation in the adult brain as well as neurogenic responses to environmental and social stimuli (Spritzer et al., 2017; see reviews in Duarte-Guterman et al., 2015; Frick et al., 2015; Holmes, 2016; Mahmoud et al., 2016; Powers, 2016; Heberden, 2017). Such results indicate that sex steroid hormones, which also vary seasonally in most vertebrates, play a critical role in modulating the processes that characterize neurogenesis both within the hippocampus and in extra-hippocampal regions. To better understand the function of neurogenesis, it will be helpful to explore sex differences in neurogenic processes while simultaneously mapping these differences onto known sex differences in seasonal physiology and behavior. For example, it is well known that in many seasonally breeding animals, males often become reproductively active and migrate to the breeding grounds prior to females (Ball and Ketterson, 2008). The mechanisms that mediate these differences in reproductive timing, however, are unknown.

In the current study, we examined if changes in cell proliferation and cell migration during the spring are associated with the seasonal life-history transition from spring breeding to migration and summer foraging in a free-ranging population of red-sided garter snakes (*Thamnophis sirtalis parietalis*) in south central Manitoba, Canada. Although many vertebrates exhibit seasonal rhythms in physiology and behavior, one of the most spectacular examples is the spring emergence of more than 30,000 garter snakes from a single hibernaculum following 8 months of winter dormancy. In late April through May, males emerge from underground dens in large numbers and immediately begin searching for mates. Similar to many vertebrates with lek-like mating aggregations (Ball and Ketterson, 2008), male snakes emerge in much larger numbers than females early in the mating season. Approximately 1–2 weeks after males first begin to emerge from the hibernaculum, females begin emerging in increasing numbers. Upon emergence, a female garter snake can be courted by up to 100 males in a single mating ball (Joy and Crews, 1985). Neither male nor female snakes eat during winter dormancy or the mating season; rather, as breeding activity wanes, snakes disperse from the den site and migrate up to 18 km to summer foraging areas, where food is abundant and competition is less fierce (Gregory and Stewart, 1975). Most female garter snakes disperse from the den within 24 h of emergence [DIL, unpublished data; also see (Shine et al., 2006)]. In contrast, many male snakes stay within the vicinity of the den for up to several weeks, searching for and courting newly emerging females (Shine et al., 2001). The factors mediating the sexually dimorphic timing of this life-history transition are unknown, but seasonal changes in neurogenesis may play a central role during migration. Importantly, red-sided garter snakes are typically faithful to their hibernaculum and return to the same den site during the fall to prepare for another cycle of winter dormancy. Thus, enhanced spatial memory during spring migration may be critical for navigating the fall return to the hibernaculum. We previously reported that patterns of cell proliferation and cell migration in the brain vary seasonally in adult male red-sided garter snakes (Maine et al., 2014b). In this study, we asked if patterns of post-embryonic cell proliferation and cell migration vary with sex or migration status during the spring mating season.

## Methods

These experiments were performed in the field with free-ranging red-sided garter snakes in the Interlake region of Manitoba, Canada. All animals were collected from 14 to 18 May 2012. Experimental protocols were approved by the Portland State University Animal Care and Use Committee and were performed under the authority of Wildlife Scientific Permit WB12691 issued by the Manitoba Department of Sustainable Development.

### Experimental design

#### Experiment 1. variation in cell proliferation related to sex and migratory status

We asked if there are sex differences in cell proliferation and/or cell migration within the brain, and if these differences are related to changes in migratory behavior of snakes during the spring mating season. Similar to Cease et al. (2007) and Dayger and Lutterschmidt (2017), we compared non-migratory snakes collected from the den site to migratory snakes collected at the beginning of their seasonal migration to the summer feeding grounds. We used a road located approximately 1 km from the den along the migration route to aid in intercepting migrating snakes.

During the spring, female snakes collected from the road typically have visible copulatory plug residue, indicating that they mated prior to migrating from the breeding grounds. We therefore compared migrating female snakes (*n* = 10) to mated females collected from the den (*n* = 11). Females were collected from actively mating pairs and held in outdoor arenas until copulation was completed; successful mating was confirmed by the presence of a mating plug in the cloaca.

Similarly, we wanted to compare courting males collected from the den (*n* = 10) to migrating males that were still exhibiting courtship behavior. During the spring, migrating males collected at the road exhibit two different behavioral phenotypes: some males continue to actively court females while others do not, presumably because they are further along in the seasonal transition to summer feeding activity (e.g., Lutterschmidt and Maine, 2014; Lucas et al., 2017). While it is not feasible to control for variation in male sexual experience in this study (i.e., it is not possible to determine whether an individual male mated previously or how many matings a male achieved prior to migration), all snakes were sexually mature and of similar body size, which suggests they were also of similar age. We used a well-established ethogram of male courtship behavior (Lutterschmidt et al., 2004; modified from Crews, 1984; Moore et al., 2000) to categorize the reproductive status of each male as courting or non-courting. Of the 22 migrating males collected from the road in this study, 10 male snakes exhibited courtship scores ≥2, behaviors that are only expressed in a reproductive context (Crews, 1984). These males were classified as “courting” and included in Experiment 1, while the remaining 12 snakes were classified as “non-courting” and reserved for Experiment 2. Thus, we examined changes in cell proliferation related to migratory status without introducing the confounding variable of differences in reproductive status.

#### Experiment 2. variation in cell proliferation related to reproductive status

We next asked if variation in cell proliferation and/or cell migration within the adult brain is associated with the seasonal life-history transition from reproductive to non-reproductive status. To address this question, we needed to distinguish changes related to migration from those related to changes in reproductive behavior. We therefore focused on the differences between reproductive and post-reproductive snakes while keeping migratory status constant. We compared cell proliferation between the 10 courting males and 12 non-courting males collected from the road during the initial stages of spring migration. To determine changes related to reproductive status in females, we collected an additional 10 females from the den immediately upon spring emergence and prior to mating. We then compared cell proliferation between these unmated females and the 11 mated females collected from the den during Experiment 1. We confirmed unmated status by verifying the absence of a mating plug in the cloaca.

### Animal housing and tissue collection

Immediately upon capture, blood samples (200 μl) were collected within 3 min using tuberculin syringes and heparinized needles. Animals were weighed and their snout-vent length (SVL) measured before they were scale clipped on the ventrum with a unique number. All animals were adult snakes with a mean SVL of 47.2 cm (±0.67 SEM) for males and 54.6 cm (±0.96 SEM) for females; these sizes are generally indicative of adult status in *T. sirtalis parietalis* (Crews et al., 1985; Conant and Collins, 1998). Snakes then received two pulse injections of 100 mg kg^−1^ body mass 5-bromo-2′-deoxyuridine (BrdU) as in Almli and Wilczynski (2007) and Maine et al. (2014b); injections were administered sequentially into two different regions of the peritoneal cavity. BrdU is a thymidine analog that is incorporated into the DNA of mitotic cells. Our previous studies indicate that injection with BrdU does not alter reproductive behavior or brain neuropeptides in male red-sided garter snakes (Maine et al., 2014b; DIL, unpublished data). Snakes were housed in semi-natural outdoor arenas (1 × 1 × 1 m) containing a hide box and water bowl. Snakes were not offered food because they do not eat during the spring mating season. Previous studies in red-sided garter snakes have demonstrated that these housing conditions do not induce significant stress responses (Moore and Mason, 2001; Cease et al., 2007; Lutterschmidt and Maine, 2014).

Four days after their initial capture, a second blood sample was collected before snakes were euthanized with a lethal overdose of 1% sodium Brevital. Male courtship behavior was assessed prior to final tissue collection. We chose this sampling regime because it allowed us to more accurately assess the behavioral phenotypes of migrating males without the influence of capture and handling immediately preceding courtship trials (Cease et al., 2007; Lutterschmidt and Maine, 2014), it maximized our chances of observing changes in cell proliferation related to the post-mating estradiol surge in females (Whittier et al., 1987a), and it optimized the labeling of newly proliferated cells by BrdU treatment (Maine et al., 2014b).

Brains were immersion-fixed in 4% paraformaldehyde in 0.1 M phosphate buffer (pH 7.2) for 16–18 h at 4°C. Tissues were then transferred to 0.1 M phosphate buffer and stored at 4°C until sectioning. Brains were cyroprotected in 30% sucrose in 0.1 M phosphate buffer and cut on a cryostat (Leica 3050S) into four alternate series of 25-μm coronal sections. Tissues were thaw-mounted onto subbed slides (Fisherbrand Superfrost Plus) and stored at −20°C. One series of tissue was used to examine differences in cell proliferation and cell migration via BrdU immunohistochemistry. Two additional tissue series were used to examine differences in arginine vasotocin and neuropeptide Y immunoreactivity among snakes, as well as the relationship between these neuropeptides and plasma steroid hormones. These data are the subject of independent analyses presented in Lucas et al. (2017).

### Immunohistochemistry

We examined potential differences in cell proliferation among snakes using BrdU immunohistochemistry methods identical to those described by Maine et al. (2014b). Briefly, antigen retrieval was performed by incubating slides in 2 N HCl in 0.1 M phosphate-buffered saline (PBS; pH 7.4) at 37°C for 30 min to denature the DNA. BrdU immunoreactivity was examined using a rat anti-BrdU antiserum (OBT0030, Accurate Chemical, Westbury, NY, USA) at a dilution of 1:5,000 in PBS containing 0.3% Triton X and 10% normal goat serum. Sections were incubated with primary antibody for 48 h at 4°C in a humid chamber. Primary antibody signal was amplified by incubation with biotinylated goat anti-rat IgG secondary antibody (BA-9400, Vector Labs Inc., Burlingame, CA, USA) diluted 1:400 followed by avidin conjugated to horseradish peroxidase (Elite ABC peroxidase kit, Vector Labs, Inc.). Primary antibody binding was visualized using 0.25 mg/ml diaminobenzidine in 0.2% hydrogen peroxide in 0.05 M Tris buffer (pH 7.2). Tissues were counterstained for 1 min in hematoxylin, dehydrated in a graded ethanol series, cleared with Citrisolv (Fisher Scientific, Pittsburgh, PA, USA) and coverslipped.

### Histology and cell quantification

**Figure 1:**
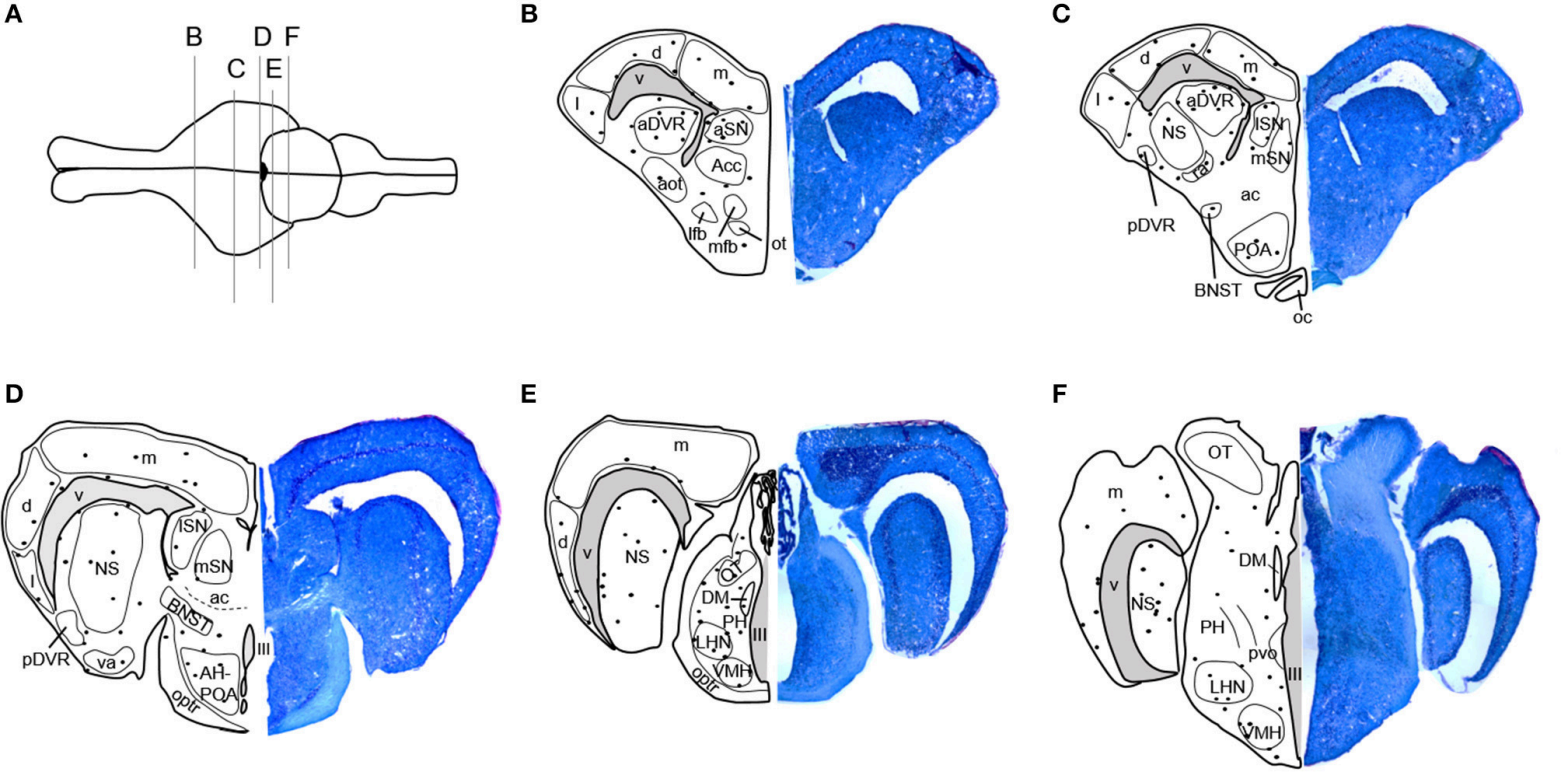
Distribution of BrdU-immunoreactive cells in the brain of adult red-sided garter snakes (*Thamnophis sirtalis parietalis*). **(A)** Schematic showing the dorsal view of the snake brain; the rostral portion of the brain is oriented to the left. The labeled vertical lines indicate the level of sections through BrdU-immunoreactive cell populations from the **(B–F)** rostral through caudal telencephalon. We quantified BrdU-labeled nuclei in the combined nucleus sphericus and anterior dorsal ventricular ridge, combined anterior preoptic area and hypothalamus, septal nucleus, and medial, dorsal, and lateral cortex. Solid black circles indicate locations of immunoreactive nuclei. Tissue sections to the right of each schematic are counterstained with toluidine blue. See Maine et al. (2014b) for a detailed atlas of BrdU-ir cells throughout the brain of red-sided garter snakes, including hindbrain structures. aot, Accessory olfactory tract; Acc, Accumbens; ac, Anterior commissure; aDVR, Anterior dorsal ventricular ridge; AH, Anterior hypothalamus; aSN, Anterior septal nucleus; BNST, Bed nucleus of the stria terminalis; d, Dorsal cortex; DM, Dorsomedial thalamic nucleus; l, Lateral cortex; lfb, Lateral forebrain bundle; LHN, Lateral posterior hypothalamic nucleus; lSN, Lateral septal nucleus; m, Medial cortex; mfb, Medial forebrain bundle; mSN, Medial septal nucleus; NS, Nucleus sphericus; III, Oculomotor nerve; ot, Olfactory tubercle; oc, Optic chiasm; OT, Optic tectum; optr, Optic tract; pvo, Paraventricular organ; PH, Periventricular hypothalamic nucleus; pDVR, Posterior dorsal ventricular ridge; POA, Preoptic area; ra, Rostral amygdaloid nucleus; va, Ventral amygdaloid nucleus; V, Ventricle; VMH, Ventromedial hypothalamic nucleus.

Stained tissue was examined using an Olympus BX40 microscope with a QIClick digital camera and QImaging software (QImaging, Surrey, Bristish Columbia, Canada). Locations of BrdU-immunoreactive (ir) cells were mapped onto standard anatomical brain sections adapted from Krohmer et al. (2010) and Martínez-Marcos et al. (2001, 2005). Newly proliferated, BrdU-ir cells were regionally distributed in the adult red-sided garter snake brain as shown in Figure 1. BrdU-ir cells occurred mainly in the ventricular zone (i.e., the ependymal cell layer of the ventricle) and the immediately adjacent tissue, especially in forebrain regions. An additional atlas and detailed description of the locations of BrdU-ir cells throughout the brain of red-sided garter snakes can be found in Maine et al. (2014b). Similar to our previous study, we quantified BrdU-ir cell number in the nucleus sphericus (NS), anterior dorsal ventricular ridge, anterior, lateral, and medial septal nucleus (SN), preoptic area, hypothalamus, and medial, dorsal, and lateral regions of the cortex. Because distinct boundaries could not be consistently established between the BrdU-ir cell populations in the NS and anterior dorsal ventricular ridge, labeled cells located in the anterior dorsal ventricular ridge were grouped into and analyzed with the NS. Similarly, distinct boundaries could not be identified between labeled nuclei in the preoptic area and hypothalamus, and thus labeled cells within these regions were combined for analysis.

**Figure 2:**
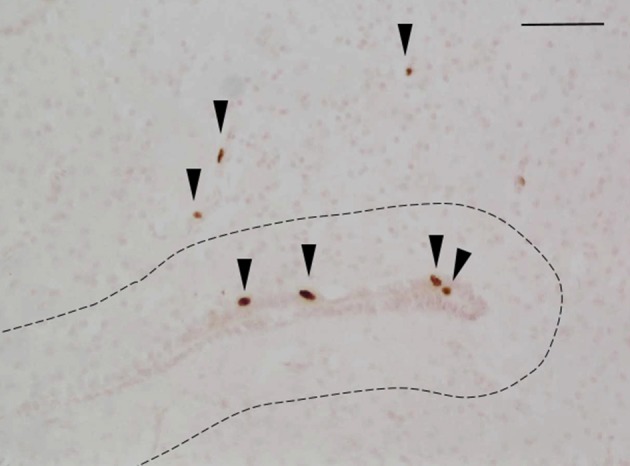
Example photomicrograph showing BrdU-labeled cells in the brain of red-sided garter snakes (*Thamnophis sirtalis parietalis*). Arrows indicate BrdU-immunoreactive nuclei; scale bar = 50 μm. The dashed line delineates a distance of 50 μm from the ependymal layer of the lateral ventricle, which was used to further categorize labeled cells as being located within the ventricular zone (within 50 μm of the ventricle) or parenchymal region (more than 50 μm from the ependymal layer of the ventricle). Arrow heads point toward the ventral region of the tissue section.

Animals were coded so that the observer was blind to the identity of individuals. Within each brain region, the total number of BrdU-ir nuclei was quantified manually in every tissue section throughout the region of interest following the methods of Maine et al. (2014b). When BrdU-ir nuclei were located within a bilateral brain region (i.e., all regions of interest except the hypothalamus), cells were quantified in one hemisphere only, as there are no significant differences in BrdU-ir cell number between hemispheres in red-sided garter snakes (Maine et al., 2014b). Labeled nuclei were further categorized by their location relative to the ventricle following the methods of Almli and Wilczynski (2009) and Maine et al. (2014b). As in these studies, we selected 50 μm as a relative boundary for categorizing cells as newly proliferating vs. migrating into the surrounding brain tissue. A BrdU-ir cell was considered to be located within the ventricular zone if it was within 50 μm of a ventricle (Figure 2). If the BrdU-labeled cell was located more than 50 μm from the ependymal layer of the ventricle it was categorized as being located within the parenchymal region (Almli and Wilczynski, 2009; Maine et al., 2014b). Cell category was also confirmed by nucleus shape, as cells located close to the ventricle have nuclei that are circular in shape, while cells migrating away from the ventricle have nuclei that are elongated and oval in shape (Figure 2; Almli and Wilczynski, 2007, 2009; Delgado-Gonzalez et al., 2011). For each region of interest, we therefore report the number of BrdU-ir cells in the ventricular zone vs. the parenchymal region.

We followed the calculations and criteria described by Laberge et al. (2008) and used in Maine et al. (2014b) to account for missing and/or damaged sections. Within each region of interest, the number of BrdU-ir cells for an individual was calculated as the total number of labeled cells divided by the total number of usable brain sections multiplied by the average number of brain sections for a given region across all animals. We calculated the total number of labeled cells in males and females separately, because body size (and therefore brain size) is sexually dimorphic. These counting methods allowed us to retain more animals in the statistical analyses, in contrast to eliminating an animal if two or more consecutive sections were missing or damaged (e.g., Lutterschmidt and Wilczynski, 2012). The mean number of missing or damaged sections across all animals and regions was 1.19 ± 0.04 SEM (5.46% ± 0.19 SEM). Any animal missing greater than 33% of its sections within a region of interest was excluded from analyses. Final sample sizes for each region of interest are listed in Tables 1, 2.

**Table 1:** Differences in SVL-corrected BrdU-positive cell number related to sex and migratory status in adult red-sided garter snakes within the nucleus sphericus (NS), hypothalamus (HYP), septal nucleus (SN), medial cortex (MC), dorsal cortex (DC), and lateral cortex (LC).

**Location of BrdU-ir cells**	**Brain region**	**Final sample sizes**	**Sex**	**Migratory status**	**Interaction**
Ventricular zone	NS	20 (10), 21 (11)	*F*_(1, 37)_ = 7.550, ***P*** = **0.009**	*F*_(1, 37)_ = 6.461, ***P*** = **0.015**	*F*_(1, 37)_ = 13.622, ***P*** < **0.001**
	HYP	19 (10), 20 (11)	*F*_(1, 35)_ = 0.226, *P* = 0.637	*F*_(1, 35)_ = 3.419, *P* = 0.073	*F*_(1, 35)_ = 10.013, ***P*** = **0.003**
	SN	20 (10), 21 (11)	*H*_(1, 37)_ = 1.262, *P* = 0.261	*H*_(1, 37)_ = 4.624, ***P*** = **0.031**	*H*_(1, 37)_ = 2.679, *P* = 0.102
	MC	20 (10), 20 (11)	*F*_(1, 36)_ = 10.947, ***P*** = **0.002**	*F*_(1, 36)_ = 3.480, *P* = 0.070	*F*_(1, 36)_ = 1.853, *P* = 0.182
	DC	20 (10), 20 (11)	*F*_(1, 36)_ = 14.472, ***P*** < **0.001**	*F*_(1, 36)_ = 1.814, *P* = 0.187	*F*_(1, 36)_ = 0.007, *P* = 0.931
	LC	20 (10), 19 (11)	*H*_(1, 35)_ = 1.519, *P* = 0.218	*H*_(1, 35)_ = 3.421, *P* = 0.072	*H*_(1, 35)_ = 1.278, *P* = 0.258
Parenchyma	NS	20 (10), 21 (11)	*F*_(1, 37)_ = 0.001, *P* = 0.976	*F*_(1, 37)_ = 0.446, *P* = 0.509	*F*_(1, 37)_ = 2.940, *P* = 0.095
	HYP	19 (10), 20 (11)	*F*_(1, 35)_ = 7.059, ***P*** = **0.012**	*F*_(1, 35)_ = 3.514, *P* = 0.069	*F*_(1, 35)_ = 34.405, ***P*** < **0.001**
	SN	20 (10), 21 (11)	*F*_(1, 37)_ = 2.204, *P* = 0.146	*F*_(1, 37)_ = 1.596, *P* = 0.214	*F*_(1, 37)_ = 3.452, *P* = 0.071
	MC	20 (10), 20 (11)	*F*_(1, 36)_ = 0.526, *P* = 0.473	*F*_(1, 36)_ = 0.585, *P* = 0.449	*F*_(1, 36)_ = 0.371, *P* = 0.546
	DC	20 (10), 20 (11)	*H*_(1, 36)_ = 0.501, *P* = 0.479	*H*_(1, 36)_ = 0.585, *P* = 0.444	*H*_(1, 36)_ = 0.050, *P* = 0.823
	LC	20 (10), 19 (11)	*F*_(1, 35)_ = 3.418, *P* = 0.073	*F*_(1, 35)_ = 0.097, *P* = 0.757	*F*_(1, 35)_ = 2.683, *P* = 0.110

*Labeled cells were categorized as newly proliferated if they were found within the ventricular zone of each region of interest and migrating if they were found within the parenchymal region more than 50 μm from the ventricular zone. Sample sizes are given as total number of males (non-migrating males collected from the den), total number of females (non-migrating females collected from the den). All statistics are the main effects of factors from a general linear model multifactor ANOVA (F statistics) or a non-parametric Scheirer-Ray-Hare extension of a Kruskal-Wallis ANOVA (H statistics) within each region of interest. Statistically significant P-values are highlighted in bold font*.

**Table 2:** Variation in BrdU-labeled cell number related to reproductive status in adult red-sided garter snakes within the nucleus sphericus (NS), hypothalamus (HYP), septal nucleus (SN), medial cortex (MC), dorsal cortex (DC), and lateral cortex (LC).

**Comparison**	**Brain region**	**Final sample sizes**	**Proliferating cells in the ventricular zone**	**Migrating cells in the parenchymal region**
Courting vs. non-courting males	NS	10, 12	*t* = −2.082, ***P*** = **0.050**	*t* = −0.668, *P* = 0.512
	HYP	9, 10	*t* = −0.963, *P* = 0.349	*t* = −2.349, ***P*** = **0.031**
	SN	10, 11	*t* = −1.172, *P* = 0.256	*U* = 51.50, *P* = 0.828
	MC	10, 11	*t* = 3.201, ***P*** = **0.005**	*t* = 1.881, *P* = 0.075
	DC	10, 11	*U* = 27.00, ***P*** = **0.040**	*U* = 23.00, ***P*** = **0.016**
	LC	10, 11	*U* = 40.00, *P* = 0.280	*U* = 46.00, *P* = 0.476
Unmated vs. mated females	NS	10, 11	*t* = 0.334, *P* = 0.742	*U* = 32.50, *P* = 0.121
	HYP	10, 11	*t* = 0.082, *P* = 0.936	*t* = 0.674, *P* = 0.508
	SN	8, 11	*U* = 28.50, *P* = 0.152	*t* = 0.916, *P* = 0.372
	MC	10, 9	*U* = 37.00, *P* = 0.495	*t* = −0.449, *P* = 0.659
	DC	10, 11	*U* = 47.00, *P* = 0.507	*t* = 1.467, *P* = 0.159
	LC	10, 11	*U* = 40.00, *P* = 0.094	*t* = 1.164, *P* = 0.259

*Labeled cells were categorized as newly proliferated if they were found within the ventricular zone of each region of interest and migrating if they were found within the parenchymal region more than 50 μm from the ventricular zone. Final sample sizes are given as courting, non-courting males collected from the road and unmated, mated females collected from the den. All statistics are from a t-test (t statistics) or non-parametric rank sum test (U statistics) within each region of interest. Statistically significant P-values are highlighted in bold font*.

### Statistical analyses

Data were transformed where necessary to meet the assumptions of parametric analysis. All data exhibited equal variance among groups. When data transformation could not correct for non-normality, we used a non-parametric Mann*-*Whitney *U*-test or a Scheirer-Ray-Hare extension of the Kruskal-Wallis ANOVA. We used SigmaPlot 12.0 (Systat Software) for all statistical analyses.

In Experiment 1, we first used *t*-tests within each sex to confirm that body size did not differ between den- and road-collected snakes. Using the same individual snakes included in this study, Lucas et al. (2017) showed that the volume of both the medial cortex and NS are positively related to the SVL of snakes. Thus, SVL can be used as a proxy for brain region volume in red-sided garter snakes. Because body size is sexually dimorphic in this population, with females being larger than males, we corrected our cell counts for potential sex differences in brain volume by dividing the total number of ir cells for each individual by its SVL. We then compared these SVL-corrected data using a general linear model two-way ANOVA with sex and migratory status as between-subjects factors for each brain region of interest. Significant main effects from these analyses were further examined using Tukey's multiple comparisons tests.

In Experiment 2, we used *t*-tests to confirm that body size of snakes did not differ between reproductive states. We then used a *t*-test within each region of interest to compare BrdU-ir cell number between reproductive conditions: courting and non-courting males collected from the road and mated and unmated females collected from the den. Data were not corrected for SVL because these analyses compared cell proliferation and cell migration within each sex, and there was very little variation in SVL among either male (mean ± SEM: 47.2 ± 0.67 cm) or female (54.6 ± 0.96 cm) snakes. These comparisons allowed us to assess potential differences in cell proliferation and cell migration related to changes in reproductive behavior while holding migratory status constant.

## Results

Body size (snout-vent-length) did not vary significantly between migrating snakes collected from the road and non-migrating snakes collected from the den for either males (*t* = 1.336, *df* = 18, *P* = 0.198) or females (*t* = 0.844, *df* = 19, *P* = 0.409). Similarly, there were no significant differences in SVL between courting and non-courting males (*t* = 0.058, *df* = 20, *P* = 0.954) or unmated and mated females (*t* = 1.423, *df* = 19, *P* = 0.171).

### Experiment 1. variation in cell proliferation related to sex and migratory status

**Figure 3:**
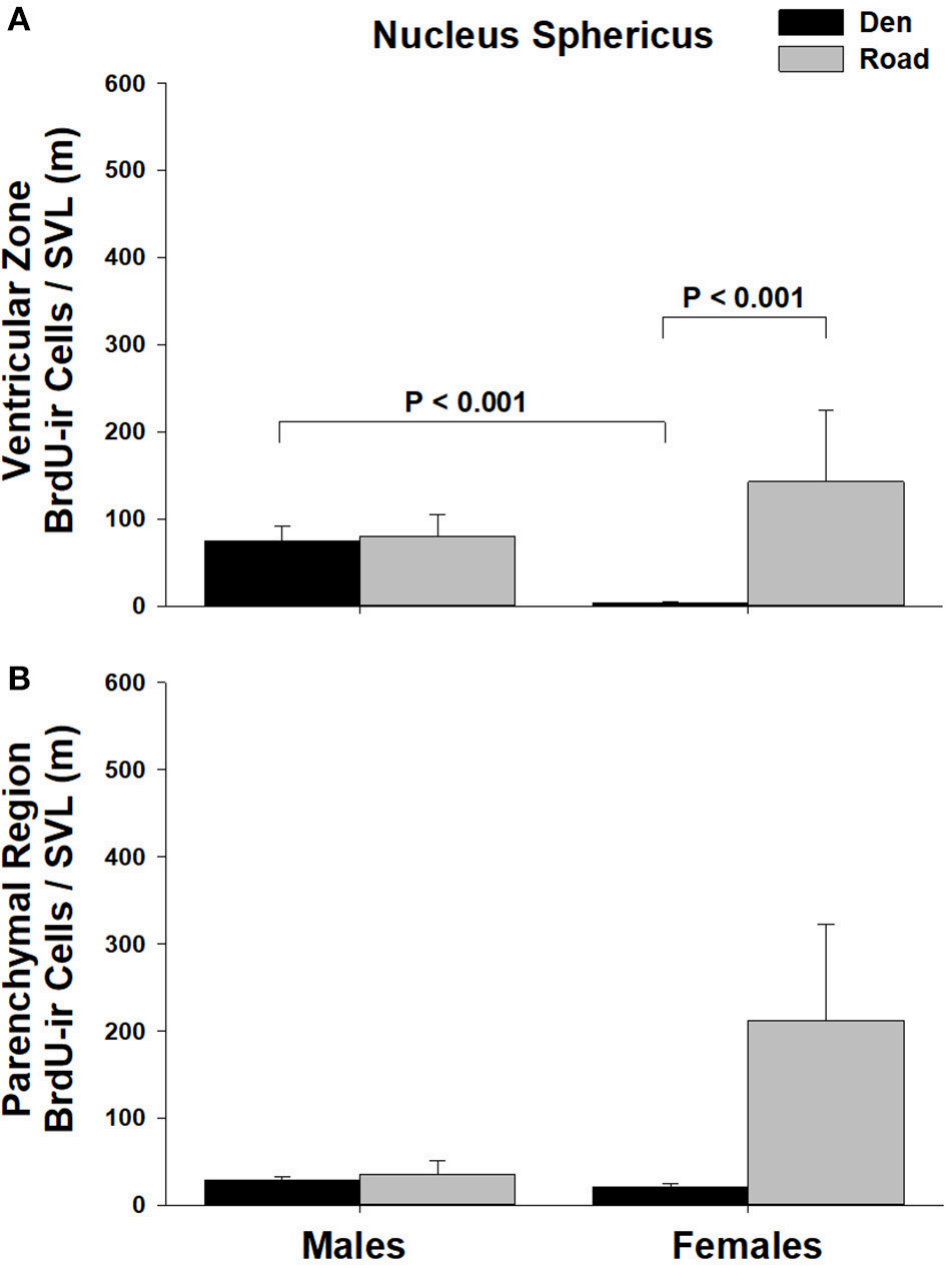
Effects of sex and migratory status on the number of BrdU-immunoreactive (ir) cells in the **(A)** ventricular zone or **(B)** parenchymal region of the nucleus sphericus (NS) in red-sided garter snakes (*Thamnophis sirtalis parietalis*). We captured snakes from the den prior to migration from the breeding grounds; migratory snakes were intercepted on a road located 1 km from the den along the migratory route. Similar to Lucas et al. (2017), we corrected BrdU-ir cell number for sex differences in body size by dividing the number of labeled cells within each individual by its snout-vent length (SVL). Each bar is the mean number of labeled cells +1 SEM. Statistics are from multiple comparisons tests.

**Figure 4:**
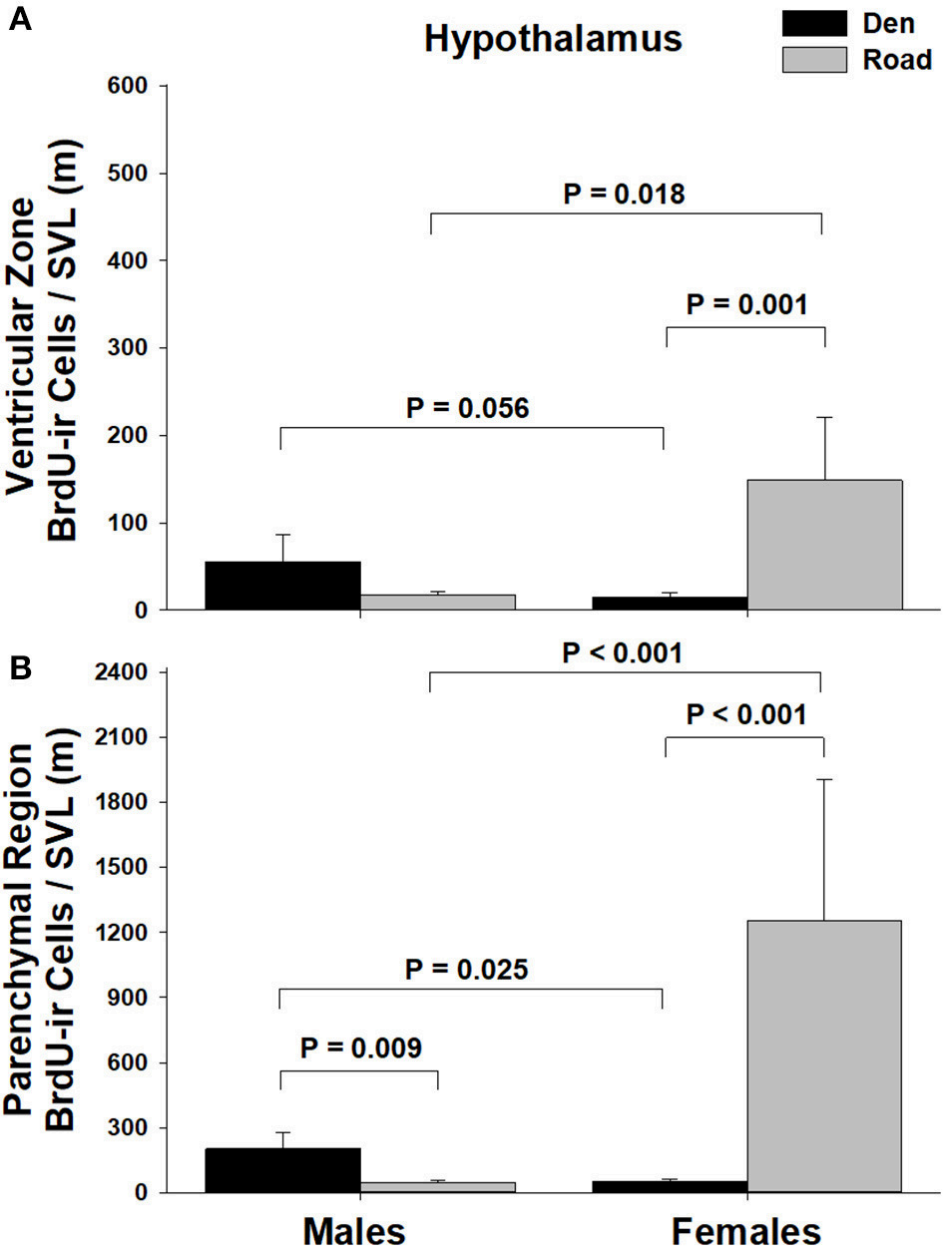
Effects of sex and migratory status on the number of BrdU-immunoreactive (ir) cells in the **(A)** ventricular zone or **(B)** parenchymal region of the hypothalamus in red-sided garter snakes (*Thamnophis sirtalis parietalis*). We captured snakes from the den prior to migration from the breeding grounds; migratory snakes were intercepted on a road located 1 km from the den along the migratory route. Similar to Lucas et al. (2017), we corrected BrdU-ir cell number for sex differences in body size by dividing the number of labeled cells within each individual by its snout-vent length (SVL). Each bar is the mean number of labeled cells + 1 SEM. Statistics are from multiple comparisons tests.

**Figure 5:**
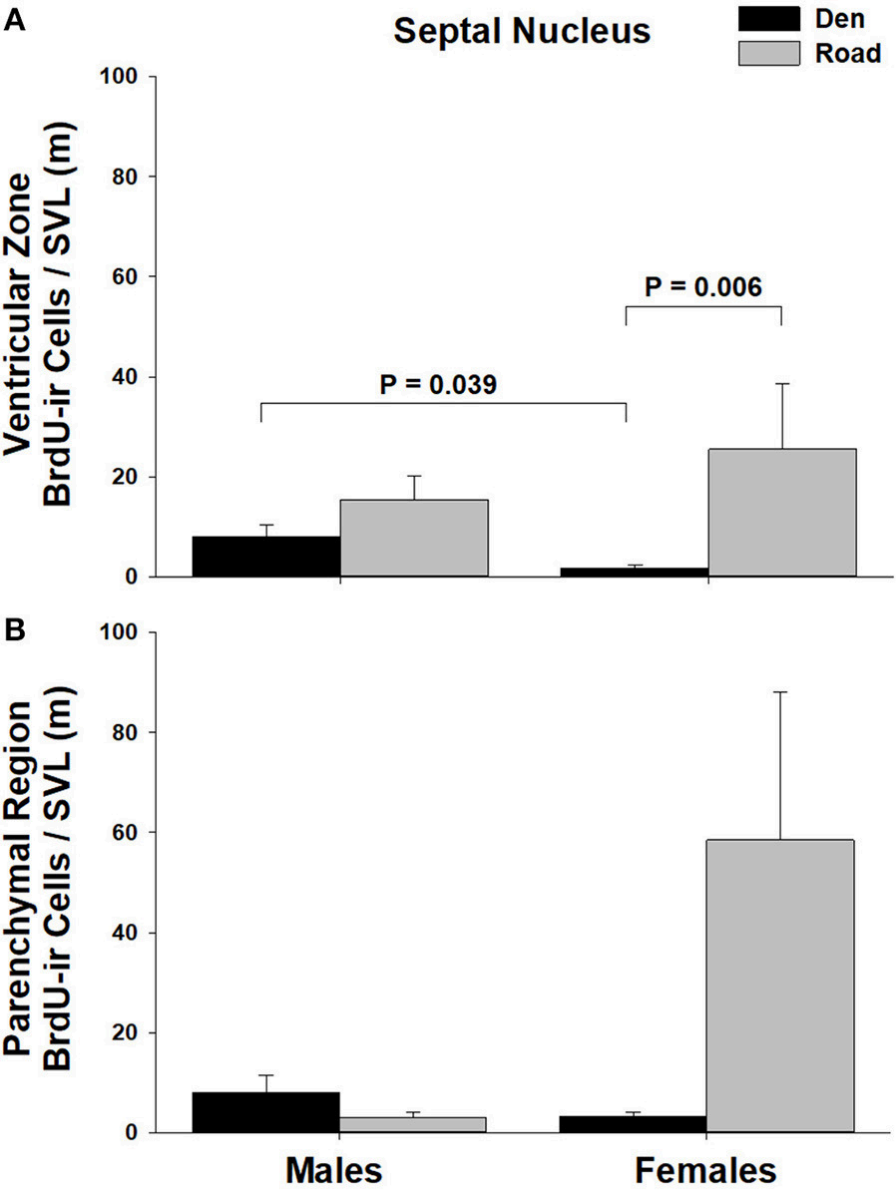
Effects of sex and migratory status on the number of BrdU-immunoreactive (ir) cells in the **(A)** ventricular zone or **(B)** parenchymal region of the septal nucleus (SN) in red-sided garter snakes (*Thamnophis sirtalis parietalis*). We captured snakes from the den prior to migration from the breeding grounds; migratory snakes were intercepted on a road located 1 km from the den along the migratory route. Similar to Lucas et al. (2017), we corrected BrdU-ir cell number for sex differences in body size by dividing the number of labeled cells within each individual by its snout-vent length (SVL). Each bar is the mean number of labeled cells +1 SEM. Statistics are from multiple comparisons tests.

**Figure 6:**
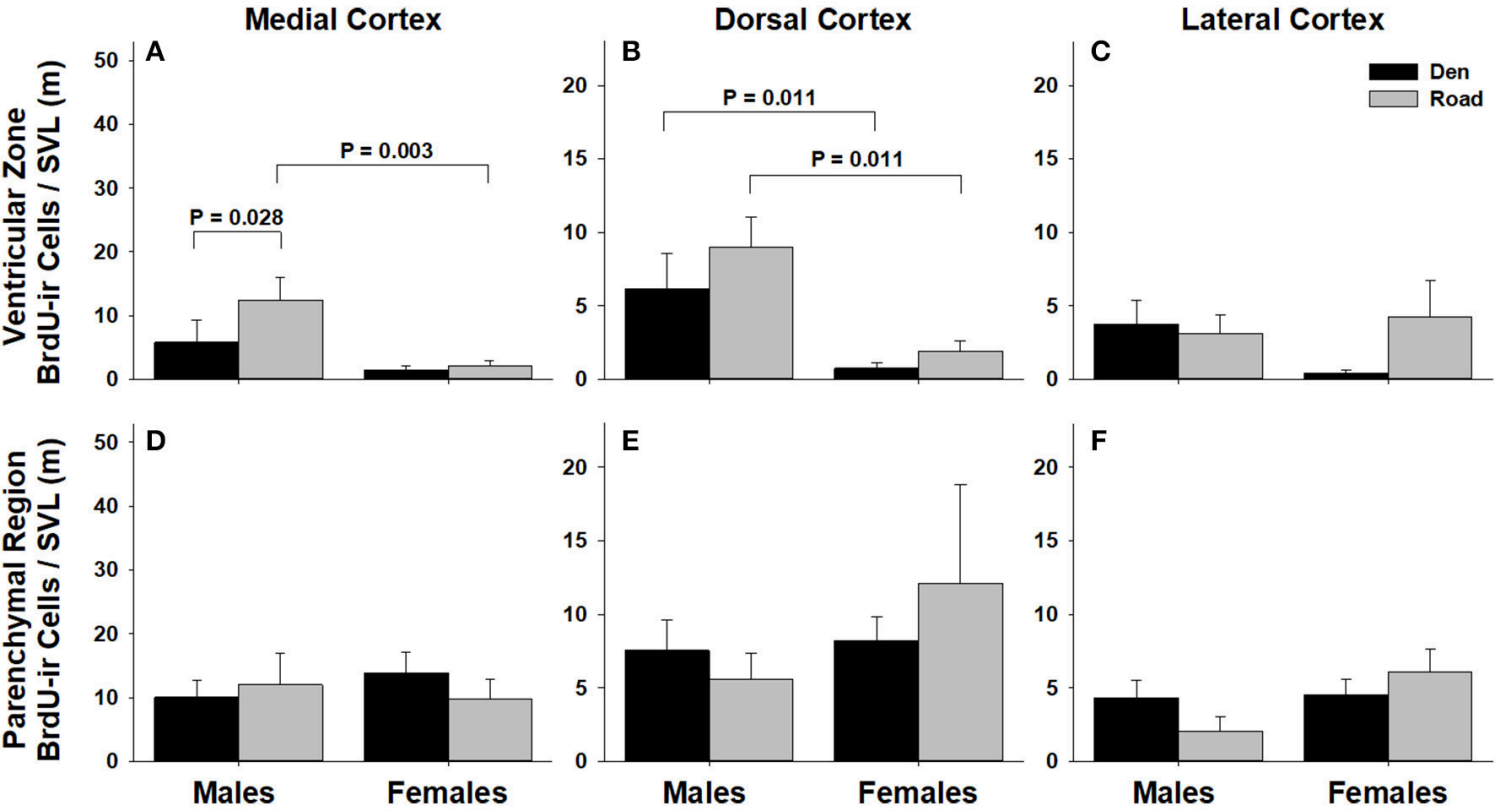
Effects of sex and migratory status on the number of BrdU-immunoreactive (ir) cells in the ventricular zone or parenchymal region of the medial cortex **(A,D)**, dorsal cortex **(B,E)**, and lateral cortex **(C,F)** in red-sided garter snakes (*Thamnophis sirtalis parietalis*). We captured snakes from the den prior to migration from the breeding grounds; migratory snakes were intercepted on a road located 1 km from the den along the migratory route. Similar to Lucas et al. (2017), we corrected BrdU-ir cell number for sex differences in body size by dividing the number of labeled cells within each individual by its snout-vent length (SVL). Each bar is the mean number of labeled cells +1 SEM. Statistics are from multiple comparisons tests.

Table 1 summarizes the effects of sex and migratory status on newly proliferated and migrating cells within each brain region of interest; results from multiple comparisons tests, where appropriate, are shown in Figures 3–6. The number of BrdU-labeled cells observed in the ventricular zone varied significantly with the main effects of sex in the NS (Figure 3A) and the medial and dorsal cortex (Figures 6A,B). Males had more labeled cells than females in each region, even after accounting for sex differences in snout-vent length. Within the NS and SN (Figures 3A, 5A), migrating snakes collected from the road had significantly more BrdU-ir cells in the ventricular zone than non-migrating snakes collected from the den. However, we observed a significant interaction between sex and migratory status within the NS as well as the hypothalamus (Figures 3A, 4A), indicating that the sex differences observed in the number of BrdU-labeled cells within the ventricular zone of these regions depended on the snakes' migratory status.

In all regions of interest except the hypothalamus, the number of migrating BrdU-labeled cells observed in the parenchymal region did not vary significantly with sex or the migratory status of snakes (Table 1). All interactions between factors in these regions were also statistically non-significant. Within the hypothalamus, the number of BrdU-ir cells in the parenchymal region varied significantly with sex, and there was a statistically significant interaction between sex and the migratory status of snakes (Figure 4B).

### Experiment 2. variation in cell proliferation related to reproductive status

**Figure 7:**
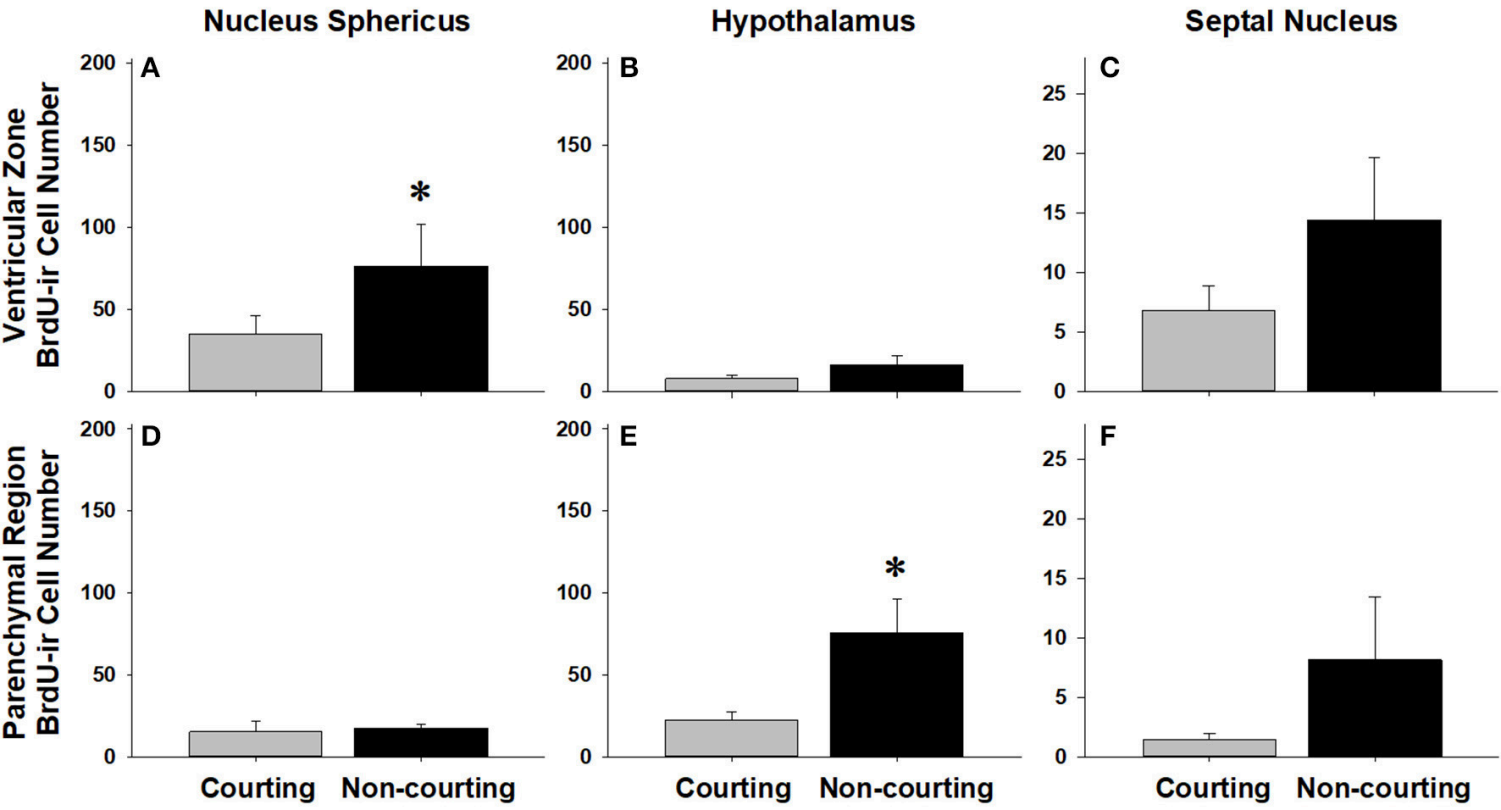
Influence of male reproductive status on the number of BrdU-immunoreactive (ir) cells in the ventricular zone or parenchymal region of the nucleus sphericus **(A,D)**, hypothalamus **(B,E)**, and septal nucleus **(C,F)** in red-sided garter snakes (*Thamnophis sirtalis parietalis*). Both courting and non-courting males were collected from the road after they had dispersed from the den to control for possible differences in migratory status. Each bar is the mean number of labeled cells +1 SEM. Asterisks indicate significant differences in BrdU cell number between courting and non-courting males.

**Figure 8:**
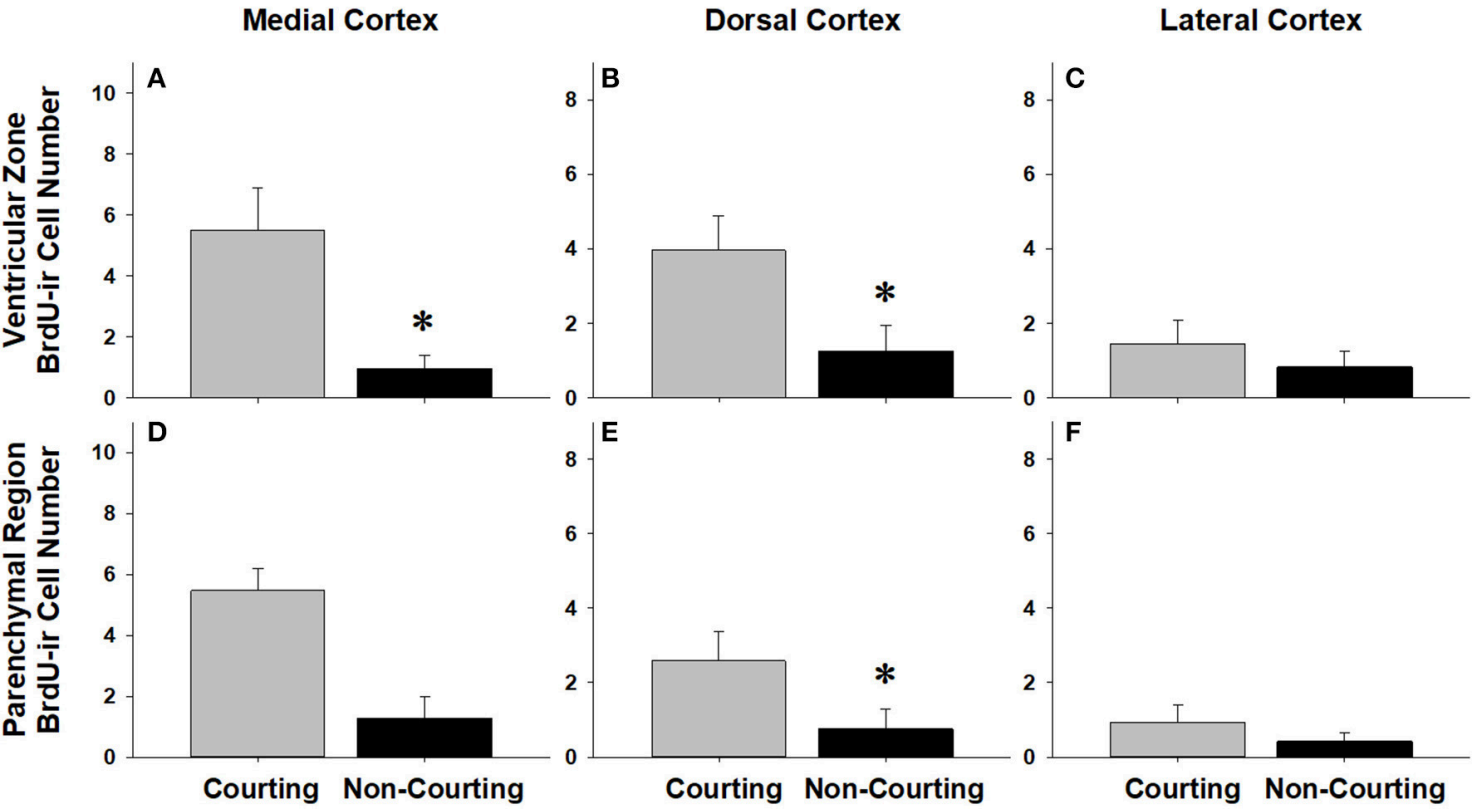
Influence of male reproductive status on the number of BrdU-immunoreactive (ir) cells in the ventricular zone or parenchymal region of the medial cortex **(A,D)**, dorsal cortex **(B,E)**, and lateral cortex **(C,F)** in red-sided garter snakes (*Thamnophis sirtalis parietalis*). Both courting and non-courting males were collected from the road after they had dispersed from the den to control for possible differences in migratory status. Each bar is the mean number of labeled cells +1 SEM. Asterisks indicate significant differences in BrdU cell number between courting and non-courting males.

We asked if changes in BrdU-ir cell number in the brain are related to the transition from courting to non-courting in males or from unmated to mated status in females. Table 2 and Figures 7, 8 show the effects of reproductive status on the number of newly proliferated and migrating cells within each brain region of interest. In summary, non-courting males had significantly more BrdU-ir cells in the ventricular zone of the NS (Figure 7A) and the parenchymal region of the hypothalamus (Figure 7E) compared to courting males. In contrast, BrdU-labeled cell number was significantly lower in non-courting males within the ventricular zone of the medial (Figure 8A) and dorsal (Figure 8B) cortex as well as the parenchymal region of the dorsal cortex (Figure 8E). In female snakes, BrdU-labeled cell number within the ventricular zone and parenchymal region did not vary significantly with reproductive status in any brain region of interest (Table 2; data not shown).

## Discussion

We found that cell proliferation and cell migration in the adult brain of red-sided garter snakes varied significantly with sex, the migratory status of snakes, and reproductive behavior in males. Furthermore, the patterns of these differences varied among the regions of interest. Four general patterns emerge from these data: (1) Prior to migration from the den site, male snakes exhibited higher levels of cell proliferation in the ventricular zone of all brain regions compared to females (but this difference was not statistically significant in the medial and lateral cortex); (2) Compared to females collected from the den, migrating female snakes exhibited a statistically significant increase in cell proliferation within the ventricular zone of all brain regions except the cortex (all subregions), whereas migrating males exhibited a significant increase in cell proliferation within the medial cortex but no other brain region; (3) The only brain region where cell migration into the parenchymal region varied significantly with sex or migratory status was the hypothalamus; and (4) In females, changes in cell proliferation and cell migration during dispersal from the breeding grounds were unrelated to changes in reproductive status, whereas in males changes in cell proliferation and cell migration were observed in response to both dispersal as well as changing reproductive behavior.

An important feature of this study is the inclusion of both hippocampal and extra-hippocampal regions of interest, which will be necessary for expanding our understanding of the function and regulation of neurogenesis beyond spatial learning and memory. As in Maine et al. (2014b), we quantified cell proliferation and cell migration within the NS, hypothalamus, SN, and cortex. Similar to other vertebrates, the NS, hypothalamus, and SN are involved in regulating reproductive behavior in red-sided garter snakes: the NS and hypothalamus contain sex steroid-concentrating neurons (Halpern et al., 1982); the NS and POA/hypothalamus exhibit seasonal changes in aromatase activity (Krohmer et al., 2010); and lesions to the NS, SN, and POA/hypothalamus prior to winter dormancy alter the courtship behavior of male snakes (Krohmer and Crews, 1987a,b). Results from the latter studies suggest that the NS and SN inhibit sexual behavior, while the POA/hypothalamus facilitates reproductive behavior. In addition to its role in reproduction, the NS of reptiles is a homolog of the mammalian amygdala (Ubeda-Bañon et al., 2011) and receives input directly from vomeronasal projections (Wang and Halpern, 1988; Lanuza and Halpern, 1997, 1998). Together, the vomeronasal-NS system mediates reproductive responses of male garter snakes to the female sexual attractiveness pheromone (Mason et al., 1989) and responses of both sexes to food chemical cues. Finally, the medial cortex is a structural and functional homolog of the mammalian hippocampus; the dorsal cortex plays a similar role but to a lesser extent (Butler and Hodos, 2005). We quantified the number of BrdU-labeled cells within each cortical subregion separately to better understand if changes in cell proliferation are associated with the spatial memory required for migration. Our data suggest that sexually dimorphic changes in cell proliferation and cell migration in the adult brain contribute to sex differences in the seasonal life-history transition from reproduction to migration and foraging behavior in red-sided garter snakes.

### Sexually dimorphic cell proliferation in the brain of breeding snakes

Even after correcting for sex differences in body length, male snakes collected from the den site had significantly more newly proliferated cells within the ventricular zone of the NS, hypothalamus, SN, and dorsal cortex compared to females. This trend was also present in the medial and lateral portions of the cortex, but these differences were not statistically significant. The observed sex differences were specific to premigratory snakes: after snakes dispersed from the den, migration-related changes in cell proliferation altered the patterns of sex differences. For example, migrating females had significantly more BrdU-labeled cells in the hypothalamus than migrating males (discussed in the next section). We suggest that sex differences in cell proliferation prior to migration are associated with temporal differences in spring emergence and dispersal from the hibernaculum between sexes.

As in most studies of male red-sided garter snakes, it is not possible to determine how much time elapsed between the emergence of each individual male and its collection. In contrast, premigratory females are collected immediately upon or very soon after spring emergence, because they typically disperse from the den within 24 h. Thus, it is extremely likely that the duration of terrestrial activity (and therefore acclimation to environmental cues such as temperature) prior to collection differed between sexes. This difference may be associated with the higher levels of cell proliferation observed within the ventricular zone of males. There is precedence for this hypothesis in previous studies of baseline glucose concentrations and oxygen consumption in red-sided garter snakes (Crews et al., 1987; Maine et al., 2014a). For example, male snakes collected from the den had significantly higher baseline glucose concentrations than females. The authors hypothesized that the sex difference was related to differences in the timing of emergence and the duration of spring activity prior to collection. In male red-sided garter snakes, Maine et al. (2014b) reported that neither cell proliferation nor cell migration into the parenchyma changed over time during the spring mating season. However, changes in cell proliferation during and immediately following spring emergence have not been investigated in either sex. Such studies are necessary to determine if males also exhibit low rates of cell proliferation immediately upon spring emergence, and whether rates of cell proliferation increase during the first several days post-emergence. For example, it is likely that acclimation to spring-like environmental cues and/or increased motor activity resulting directly from spring emergence itself are associated with increases in both metabolic and mitotic processes. Such results would suggest that the observed differences in cell proliferation are a result of, rather than the cause of, sex differences in the timing of spring emergence.

The processes that characterize neurogenesis are subject to hormonal regulation, and therefore the influence of sex steroid hormones (or other sexually dimorphic endocrine factors, including glucocorticoid “stress” hormones) on cell proliferation and migration may contribute to the sex differences reported here (see reviews in Balthazart and Ball, 2016; Dunlap, 2016; Mahmoud et al., 2016; Powers, 2016). For example, treatment of territorial, but not non-territorial male side-blotched lizards (*Uta stansburiana*) with testosterone decreased the volume of the medial cortex and reduced the number of doublecortin-positive cells, an indicator of immature, migrating neurons (LaDage et al., 2017). Pellegrini et al. (2007) showed that radial glial cells in the zebrafish forebrain express aromatase, the enzyme that synthesizes estrogens from androgens. The mitotic rate of these progenitor cells as well as the migration of their newly-formed daughter cells are, surprisingly, inhibited by treatments that elevate brain estrogen levels (Diotel et al., 2013). Whether aromatase expression in radial glial cells is unique to teleosts or a conserved feature of vertebrates is not yet clear (Coumailleau et al., 2015). Nevertheless, these and other studies demonstrate that sex steroids contribute to both sex and seasonal variation in neurogenesis.

Because red-sided garter snakes exhibit a temporally dissociated reproductive pattern (reviewed in Lutterschmidt, 2012), the observed sex differences in cell proliferation and migration may not be mediated by sex steroids. For example, plasma androgens are low and declining during breeding in male snakes (e.g., Crews et al., 1984; Krohmer et al., 1987; Whittier et al., 1987b; Moore et al., 2001; Lutterschmidt and Mason, 2009), and estradiol concentrations of unmated females are low or undetectable during the mating season (Whittier et al., 1987a; Dayger et al., 2013; Dayger and Lutterschmidt, 2016, 2017). Although females exhibit an estradiol surge post-mating (Whittier et al., 1987a), we did not observe differences in cell proliferation or cell migration between unmated and mated females. It is possible that either the sex differences observed here are independent of gonadal sex steroid hormones, or that the sex differences in seasonal neurogenesis are established during winter dormancy, when plasma sex steroid hormones are elevated in both males and females in response to low temperature dormancy (Lutterschmidt and Mason, 2009). Neither of these hypotheses exclude the possibility that brain-derived neurosteroids mediate sex differences in cell proliferation during winter dormancy and/or spring mating. The timecourse of neurogenic changes during winter dormancy has not yet been characterized in any ectothermic vertebrate (but see Cerri et al., 2009), and such data will be necessary for evaluating these hypotheses.

### Sex differences in cell proliferation related to snake migration

After dispersing from the den during the spring mating season, migrating females collected from the road showed a statistically significant increase in cell proliferation within the ventricular zone of the NS, hypothalamus, and SN. In contrast, cell proliferation within these same brain regions did not vary significantly with migratory status in males. These observations support and expand upon the hypothesis described in the previous section: females exhibit low levels of cell proliferation compared to males immediately post-emergence, and cell proliferation increases once females have been terrestrially active for a period of time comparable to that of males. Alternatively, it is possible that sexually dimorphic changes in cell proliferation during migration are associated with concomitant changes in other behaviors, such as reproductive or foraging behaviors. For example, most migrating male snakes continue to court females even as they migrate away from the breeding grounds (Lutterschmidt and Maine, 2014; Lucas et al., 2017), and cell proliferation within the NS of males varied significantly with reproductive status but not migratory status. Similarly, increased cell proliferation within the NS, hypothalamus, and/or SN of females after dispersal from the den may be related to the activation of foraging behavior. Preliminary data suggest that a significant proportion of females pursue feeding opportunities and eat soon after emerging from winter dormancy (DIL, unpublished data). This is in stark contrast to males, which will refuse food in lieu of courtship opportunities for up to several weeks post-emergence (Crews et al., 1987; O'Donnell et al., 2004; Lutterschmidt and Maine, 2014), even as they begin migrating to the feeding grounds (Lucas et al., 2017; DIL, unpublished data). Evaluating which of these hypotheses best explains the sex differences in cell proliferation after dispersal will require a detailed timecourse focused on the later stages of migration in both sexes. It is also possible that sex differences in navigational strategies are associated with different patterns of spatial memory development and spatial learning (e.g., Roth and Krochmal, 2015), which could in turn contribute to the observed sex differences in cell proliferation and cell migration. The navigational mechanisms utilized by red-sided garter snakes during migration to and from the hibernaculum are not yet known.

Surprisingly, there were no migration-related changes in cell proliferation within any subregion of the cortex in females, whereas the only migration-related change in cell proliferation observed in males occurred within the medial cortex. The reptilian medial cortex is a structural and functional homolog of the avian and mammalian hippocampus (Butler and Hodos, 2005), and variation in cortical neurogenesis and/or volume is positively correlated with territory and home range size in reptiles (Roth et al., 2006; LaDage et al., 2009; Powers, 2016). While it is possible that the increase in cell proliferation within the medial cortex of migrating males is related to changing spatial learning and memory demands, it is unlikely that such a change would be sexually dimorphic, as females also migrate similar distances to summer feeding areas. We speculate that the significant increase in cell proliferation within the medial cortex of migrating males is involved in regulating changes in the sensitivity of the hypothalamic-pituitary-adrenal (HPA) axis during the spring. It is well established that male red-sided garter snakes exhibit decreased sensitivity to capture stress during the mating season (e.g., Moore et al., 2000, 2001; Lutterschmidt and Mason, 2005; Cease et al., 2007). Further, breeding males are less sensitive to adrenocorticotrophic hormone (ACTH) challenge compared to both females and fall-collected males (Dayger and Lutterschmidt, 2016). One possible explanation for decreased activity of the HPA axis is a change in its negative feedback regulation by the hippocampus. For example, neuropeptide Y (NPY)-positive cells within the hippocampus have a neuromodulatory influence on the sensitivity of the HPA axis in rats (Thorsell et al., 2000; Heilig and Thorsell, 2002; Morales-Medina et al., 2010; Schneider et al., 2013). Intriguingly, we observed sexually dimorphic changes in NPY-positive cell number in the cortex of the same animals used in this study: non-courting males had significantly more NPY-labeled cells in the cortex compared to courting males, whereas females showed no change in NPY-labeled cell number in relation to either changing migratory or reproductive status (Lucas et al., 2017). It is possible that increased cell proliferation within the medial cortex of migrating males contributes to the seasonal changes in cortical NPY cell number reported in Lucas et al. (2017), and that changing NPY cell number in turn mediates seasonal changes in HPA axis sensitivity and/or activity. Studies using double-labeling techniques to determine the fate of newly proliferated, BrdU-labeled cortical cells are necessary to determine this possibility. It would also be interesting to test if blocking cell proliferation pharmacologically during spring migration alters the sensitivity of the HPA axis.

### Regional variation in cell migration into the parenchyma

In the adult brain, new cells are born from dividing radial glial cells located within the ependymal layer of the ventricles. These newly-born cells are undifferentiated and can develop into either neurons or glial cells as they migrate to their final destination in the parenchyma. Lopez-Garcia et al. (1990) reported that a minimum of 7 days is required for newly proliferated cells to differentiate into neurons in Iberian wall lizards (*Podarcis hispanica*). We observed BrdU-labeled cells within the parenchymal region 4 days after treatment with BrdU. Because our 4-day timecourse is likely not long enough for new cells to migrate into the parenchyma and/or fully differentiate into mature neurons or glia, it is possible that the presence of BrdU-positive cells within the parenchyma of all brain regions in this study results from the continued division of newly proliferated cells (i.e., transient amplification) (Delgado-Gonzalez et al., 2011). Regardless of the cause, our results indicate significant variation among brain regions in cell migration and/or continued cell division within the parenchyma during the spring mating season.

The only brain region where the number of BrdU-labeled cells within the parenchymal region varied significantly with sex or migratory status was the hypothalamus. Similar to differences in cell proliferation, males had significantly more BrdU-labeled cells in the parenchymal region compared to females prior to migration, but migrating females showed a significant increase in BrdU-labeled cell number within the parenchyma, resulting in a reversal of the sex difference. Our results suggest that cell proliferation and/or cell migration are activated earlier or to a greater extent in the hypothalamus compared to other brain regions. Because the processes of neurogenesis are influenced by environmental temperature (Powers, 2016), it is possible that the differences reported here are established during low-temperature winter dormancy. For example, in the tropical lizard *Tropidurus hispidus*, acclimation to low temperatures decreases cell migration but not cell proliferation (Marchioro et al., 2012). Similar results were reported in Iberian wall lizards (*P. hispanica*) (Ramirez et al., 1997). In red-sided garter snakes, several different neuroendocrine factors and hypothalamic circuits are known to be highly responsive to low temperature winter dormancy (Krohmer and Lutterschmidt, 2011; Lutterschmidt, 2012), and therefore temperature-induced changes in cell proliferation or cell migration within the hypothalamus could explain the differences in BrdU-labeled cell number within the parenchymal region reported here.

### Changes in cell proliferation related to reproductive status

In females, changes in cell proliferation during dispersal from the breeding grounds were unrelated to changes in reproductive status, as there were no significant differences in cell proliferation or migration between mated and unmated females in any brain region. In males, changes in cell proliferation and cell migration were observed in response to both dispersal and changing reproductive behavior. We propose that the pattern of these changes in males reflects the timecourse of neurogenesis from spring emergence and the activation of courtship behavior to migration and the cessation of reproductive behavior. For example, non-courting males exhibited more cell proliferation in the NS and more cell migration into the parenchyma of the hypothalamus than courting males. These changes mirror those observed in migrating females, suggesting that males do exhibit a similar increase in cell proliferation and cell migration during dispersal from the den, but only after courtship behavior has waned. This hypothesis may also explain why cell migration within the hypothalamus initially decreases in courting males collected from the road (Figure 4B), but subsequently increases as males transition to non-courting status (Figure 7E). Similarly, migrating males that still express courtship behavior exhibit a significant increase in cell proliferation within the medial cortex, but as migrating males transition to non-courting reproductive status, there is a subsequent and significant decrease in cell proliferation within the medial and dorsal cortex and a decrease in cell migration within the dorsal cortex (and probably the medial cortex: *P* = 0.075). Collectively, these results suggest that there is a timecourse of changes in cell proliferation and migration in males during the spring mating season, from courting females at the breeding grounds, to simultaneously migrating but continuing to court females if the opportunity arises, and finally to migrating to summer foraging areas as reproductive behavior wanes and feeding behavior is activated.

## Conclusions

In summary, we found that cell proliferation and cell migration in the adult brain of red-sided garter snakes varied significantly with sex, the migratory status of snakes, and reproductive behavior in males. Our data suggest that changes in cell proliferation and cell migration within the adult brain are involved in the seasonal life-history transition from reproduction to migration and foraging behavior in red-sided garter snakes. Moreover, differences in the observed patterns of cell proliferation and cell migration between males and females may contribute to known sex differences in the timing of this life-history transition during the spring mating season. To better understand how the processes of neurogenesis contribute to seasonal changes in physiology and behavior, future research directions should include elucidating the timecourse of neurogenic changes during multiple life-history stages, especially winter dormancy, emergence, and migration. Subsequent studies could then determine if the newly proliferated cells differentiate into primarily neurons or glial cells, as well as the neurochemical phenotype of the surviving, mature cells. Finally, knowing if and how physiology and behavior change when rates of cell proliferation and cell migration are altered pharmacologically will be critical for understanding the functional significance of post-embryonic neurogenesis to seasonal rhythms and, ultimately, reproductive fitness.

## Author contributions

DL and AL designed and conducted the experiments. AL performed the immunohistochemistry assay. AL, RK, VN, and MR quantified the immunoreactive cell data. DL and AL analyzed and interpreted the data. DL wrote the paper.

### Conflict of interest statement

The authors declare that the research was conducted in the absence of any commercial or financial relationships that could be construed as a potential conflict of interest.
